# Supplementary material for: Transforming scholarly landscapes: The influence of large language models on academic fields beyond computer science
Source: PLoS One. 2026 Jan 14;21(1):e0337127. doi: 10.1371/journal.pone.0337127 (PMC12893815; doi:10.1371/journal.pone.0337127)
Supplement: S1 Appendix — (PDF) [file pone.0337127.s001.pdf]

Appendix: Supplementary Definitions and Discussions

Choosing the LLMs

The Computer Science community has yet to establish a universally accepted definition of LLMs [1]. The term LLM gained prominence recently amidst a paradigm shift in AI, marked by the emergence of models trained on broad data, which started with textual data but soon expanded to other data modalities (such as images, codes, proteins, etc). These models belong to a class called Foundation models [2].

In our analysis, an LLM refers to an impactful foundation model [3] based on the transformer architecture pre-trained on massive textual datasets. We curate these models from the Stanford Ecosystem Graphs [4], which contains 106 foundation models pre-trained on textual data as of February 2024.

S2 Dataset Statistics and Reliability

The Semantic Scholar Dataset (S2) that we use in our analysis (which is the backbone of Semantic Scholar) covers all STM (Science, Technology, and Medicine) and SSH (Social Sciences and Humanities) disciplines, including biology, medicine, computer science, geography, business, history, and economics.

The February 2024 version of the S2 comprises approximately 200M papers metadata, sourced from diverse partners such as PubMed, Springer Nature, Taylor & Francis, SAGE, Wiley, ACM, IEEE, arXiv, and Unpaywall etc. Originally housing 84.1M papers, the dataset is continuously updated on a monthly basis. Further, each paper in the dataset is annotated with fields of study with an annotation accuracy of 86%, using a taxonomy adapted from the Microsoft Academic Graph [5].

Compared to other datasets (e.g., arXiv, Scopus, Pubmed), S2 stands out as the largest and most up-to-date open-access scholarly dataset that includes parsed text and metadata such as field of study, citation contexts, citation graph, etc. Overall, the S2 dataset is a good representative of the non-CS fields [6–9].

Examining LLM citation within CS

The main objective of this work is to assess the impact of LLMs on fields outside of CS. However, in this section, we analyze a control, LLM citations within CS, to provide a broader context for our findings. To add to this, we found that the LLMs receive $\sim 91k$ citations from within CS, which is ~ 5.5 times higher than the citations it garners from Linguistics (the field that gives the highest LLM citations outside CS). This discrepancy is expected, considering the higher popularity of LLMs within CS.

References

1. Radford A, Wu J, Child R, Luan D, Amodei D, Sutskever I, et al. Language models are unsupervised multitask learners. In: OpenAI blog. vol. 1; 2019. p. 9. Available from: `https://cdn.openai.com/better-language-models/language_models_are_unsupervised_multitask_learners.pdf`.

2. Bommasani R, Hudson DA, Adeli E, Altman R, Arora S, von Arx S, et al. On the opportunities and risks of foundation models. arXiv preprint arXiv:210807258. 2021;.

3. Henderson P, Li X, Jurafsky D, Hashimoto T, Lemley MA, Liang P. Foundation models and fair use. In: Journal of Machine Learning Research. vol. 24; 2023. p. 1–79. Available from: https://www.jmlr.org/papers/volume24/23-0569/23-0569.pdf.

4. Bommasani R, Soylu D, Liao TI, Creel KA, Liang P. Ecosystem graphs: The social footprint of foundation models. arXiv preprint arXiv:230315772. 2023;.

5. Kinney R, Anastasiades C, Authur R, Beltagy I, Bragg J, Buraczynski A, et al. The semantic scholar open data platform. arXiv preprint arXiv:230110140. 2023;.

6. Wahle JP, Ruas T, Abdalla M, Gipp B, Mohammad S. We are Who We Cite: Bridges of Influence Between Natural Language Processing and Other Academic Fields. In: Bouamor H, Pino J, Bali K, editors. Proceedings of the 2023 Conference on Empirical Methods in Natural Language Processing. Singapore: Association for Computational Linguistics; 2023. p. 12896–12913. Available from: https://aclanthology.org/2023.emnlp-main.797.

7. Wahle JP, Lima Ruas T, Abdalla M, Gipp B, Mohammad SM. Citation Amnesia: On The Recency Bias of NLP and Other Academic Fields. In: Rambow O, Wanner L, Apidianaki M, Al-Khalifa H, Eugenio BD, Schockaert S, editors. Proceedings of the 31st International Conference on Computational Linguistics. Abu Dhabi, UAE: Association for Computational Linguistics; 2025. p. 1027–1044. Available from: https://aclanthology.org/2025.coling-main.69/.

8. Illia F, Nooraeni R, Suadaa LH. Implementation of Topic Modeling in the Analysis of Topic Trends in SDGs Goal 6 Research. In: 2023 International Conference on Electrical Engineering and Informatics (ICEEI). IEEE; 2023. p. 1–6. Available from: https://ieeexplore.ieee.org/document/10346917.

9. Guo Y, Chang JC, Antoniak M, Bransom E, Cohen T, Wang L, et al. Personalized Jargon Identification for Enhanced Interdisciplinary Communication. In: Duh K, Gomez H, Bethard S, editors. Proceedings of the 2024 Conference of the North American Chapter of the Association for Computational Linguistics: Human Language Technologies (Volume 1: Long Papers). Mexico City, Mexico: Association for Computational Linguistics; 2024. p. 4535–4550. Available from: https://aclanthology.org/2024.naacl-long.255/.
