# Supplementary material for: Transforming scholarly landscapes: The influence of large language models on academic fields beyond computer science
Source: PLoS One. 2026 Jan 14;21(1):e0337127. doi: 10.1371/journal.pone.0337127 (PMC12893815; doi:10.1371/journal.pone.0337127)
Supplement: S2 Appendix — (PDF) [file pone.0337127.s002.pdf]

Appendix: Human Evaluation of LLM Citing Papers That Discuss LLM Risks

Titles and abstracts are primarily meant to provide a summary of a paper's focus, while citation contexts offer detailed insights into how the citing paper utilizes the cited work, specifically LLMs in this case. When referring to citation contexts, we mean sentences that include citation marks. More precisely, these are sentences that explicitly mention LLMs by name or citation. Hence, we use texts from these sections to filter papers that acknowledge the ethical risks of LLMs. Specifically, we look for the keywords in Table 1 in those sections. This filtering results in 1,200 research papers in 22 fields from 148,501 papers citing LLMs. Subsequently, we manually annotate the titles and citation contexts in these 1200 papers to explore how these non-CS papers acknowledge the risks pertaining to LLMs and take steps to mitigate them. We categorize citation contexts that mention LLM-related risks into two classes: "acknowledge", which denotes contexts that solely acknowledge the risks, and "mitigate", denoting contexts that make attempts to alleviate the issues posed by LLMs (examples in Table 2). Additionally, in Table 3) we provide a list of 10 titles from the papers that cite LLMs and explicitly discuss the ethical context of AI.

Table 1. Keywords to filter papers.

ethics	risks	limitations
drawbacks	bias	ethical consideration

Evaluation: To assess the reliability of the aforementioned analysis method, we conducted a manual analysis of 100 papers discussing the ethical concerns of LLMs, revealing a high recall, as only 7 papers engaged in discussions about the ethical concerns of LLMs without explicitly mentioning the LLMs. Additionally, we manually analyzed 200 papers and found no paper mentioning ethical concerns in the same context as LLMs but not discussing LLMs' ethical risks. This reveals the high reliability of our automatic method.

Table 2. Citation contexts mentioning LLM-related ethical concerns.

Field	Year	Title	Context	Class
Psychology	2021	An Evaluation of Generative Pre-Training Model-based Therapy Chatbot for Caregivers	However, researchers found that GPT-3 did not yield satisfying performance because it generated off-topic, confusing answers and had ethical issues, such as cultural biases in its responses.	acknowledge
	2023	Comparing Sentence-Level Suggestions to Message-Level Suggestions in AI-Mediated Communication	Cautious voices have warned about the ethical and social risks of harm from large language models, ranging from discrimination and exclusion to misinformation and environmental and socioeconomic harms.	acknowledge
	2023	AI Text-to-Behavior: A Study In Steerability	However, as Ray discusses, while there's a substantial promise for steerable language models, there are also crucial challenges, biases, and ethical considerations surrounding ChatGPT and similar models.	acknowledge
Medicine	2023	From Military to Healthcare: Adopting and Expanding Ethical Principles for Generative Artificial Intelligence	We can adopt this principle for the ethical use of generative AI in healthcare and ensure that human involvement is maintained when more powerful generative AI systems such as ChatGPT or clinical decision support systems are in use.	mitigate
	2023	Foundation Models in Healthcare: Opportunities, Risks & Strategies Forward	How the use of FM-based applications may exacerbate social inequalities); and raises fundamental questions about the responsible, ethical, and safe use of such technologies going forward.	acknowledge
Biology	2021	Ten future challenges for synthetic biology	The highly acclaimed natural language processing (NLP) model GPT-3 has sparked serious ethical debate due to its ability to generate highly convincing human text, even when given only a few learning points as input in a process called few-shot-learning.	acknowledge
Sociology	2022	The Ghost in the Machine has an American accent: value conflict in GPT-3	The value alignment problem is one of the more difficult areas of the field of ethical AI, but also the most critical.	acknowledge
History	2022	Unified Detoxifying and Debiasing in Language Generation via Inference-time Adaptive Optimization	Moreover, such issues are found to persist across increasing model sizes, emphasizing the urgency of developing practical methods for ethical NLG.	acknowledge
Economics	2022	Prismal view of ethics	Something along that line of thinking, but outside of ethical considerations, was done in machine learning for solving a considerable set of tasks with the same agent.	mitigate
Business	2023	On the Planning Abilities of Large Language Models - A Critical Investigation	Of particular interest to us in this paper is the thread of efforts that aim to investigate (and showcase) reasoning abilities of LLMs, including commonsense reasoning, logical reasoning, and even ethical reasoning.	acknowledge

Table 3. Paper titles explicitly mentioning ethical context in AI.

Year	Field	Title
2020	Business	Management perspective of ethics in artificial intelligence
2022	Business	Don't "research fast and break things": On the ethics of Computational Social Science
2022	Philosophy	Metaethical Perspectives on 'Benchmarking' AI Ethics
2022	Medicine	A scoping review of ethics considerations in clinical natural language processing
2023	Political Science	Ethics in conversation: Building an ethics assurance case for autonomous AI-enabled voice agents in healthcare
2023	Business	The ethical ambiguity of AI data enrichment: Measuring gaps in research ethics norms and practices
2023	Business	How to design an AI ethics board
2023	Business	Attention is not all you need: the complicated case of ethically using large language models in healthcare and medicine
2023	Philosophy	A method for the ethical analysis of brain-inspired AI
2023	Philosophy	A high-level overview of AI ethics
