# Supplementary material for: Transforming scholarly landscapes: The influence of large language models on academic fields beyond computer science
Source: PLoS One. 2026 Jan 14;21(1):e0337127. doi: 10.1371/journal.pone.0337127 (PMC12893815; doi:10.1371/journal.pone.0337127)
Supplement: S3 Appendix — (PDF) [file pone.0337127.s003.pdf]

Appendix: Additional Results

Influence of Alternate CS Method (LDA) vs. LLM beyond CS.

In this section, we extend our analysis to compare the influence of LLMs with that of Latent Dirichlet Allocation (LDA), a popular method originating from CS but gaining traction outside CS domains as well.

In our analysis, we identified three seminal papers on LDA [1–3] based on their citation counts and examined the number of citations they received from non-CS fields. In total, these papers receive 8383 citations from non-CS fields cumulatively. Specifically, we found that 13 fields contribute more than 100 citations each, while 7 fields contribute over 500 citations each, and only one field contributes more than 1000 citations. Further, we find that the Gini index of the citation distribution of the aforementioned LDA papers among non-CS fields is 0.59 (higher than that of LLMs). From this analysis, we infer that LLMs are more popular in fields outside CS than LDA.

Usage Patterns of LLMs in non-CS Fields: Inference vs. Fine-Tuning

We investigated the extent to which papers in non-CS fields utilize LLMs through fine-tuning or solely for inference in zero-shot settings. Specifically, we searched for the keywords "fine-tune," "zero-shot," and "inference" within the abstracts of the non-CS papers that cite LLMs. This filtering process resulted in 3675 papers. Further, we uniformly sampled and manually analyzed 50 papers out of 479 papers that mentioned fine-tuning, revealing an 86% precision. Similarly, we sampled 100 papers from the remaining 3196 papers (that did not contain the word "fine-tune" in their abstracts, and 3158 of these papers contained "zero-shot" in their abstracts) and manually analyzed them, finding a 79% precision rate in representing papers that use LLMs solely for inference.

This analysis is an approximate indicator of the use of LLMs in non-CS fields, indicating that while some of the non-CS papers indeed fine-tine LLMs on domain-specific datasets, approximately 6.6 times more papers solely use LLMs for inference purposes.

References

1. Blei DM, Ng AY, Jordan MI. Latent dirichlet allocation. In: Journal of machine Learning research. vol. 3; 2003. p. 993–1022. Available from: https://www.jmlr.org/papers/volume3/blei03a/blei03a.pdf.

2. Blei DM. Probabilistic topic models. vol. 55. ACM New York, NY, USA; 2012. p. 77–84. Available from: https://dl.acm.org/doi/10.1145/2133806.2133826.

3. Teh Y, Jordan M, Beal M, Blei D. Sharing clusters among related groups: Hierarchical Dirichlet processes. vol. 17; 2004.Available from: https://papers.nips.cc/paper_files/paper/2004/file/fb4ab556bc42d6f0ee0f9e24ec4d1af0-Paper.pdf.

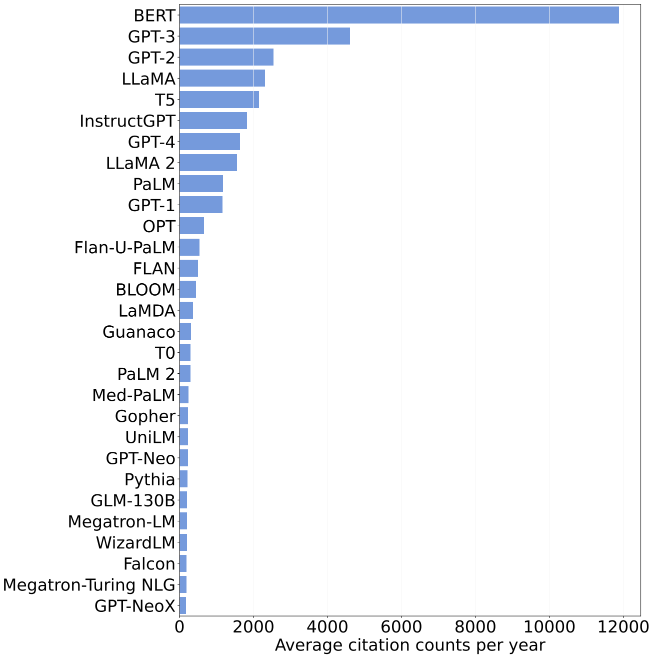

Fig 1. LLM Popularity across all non-CS fields.

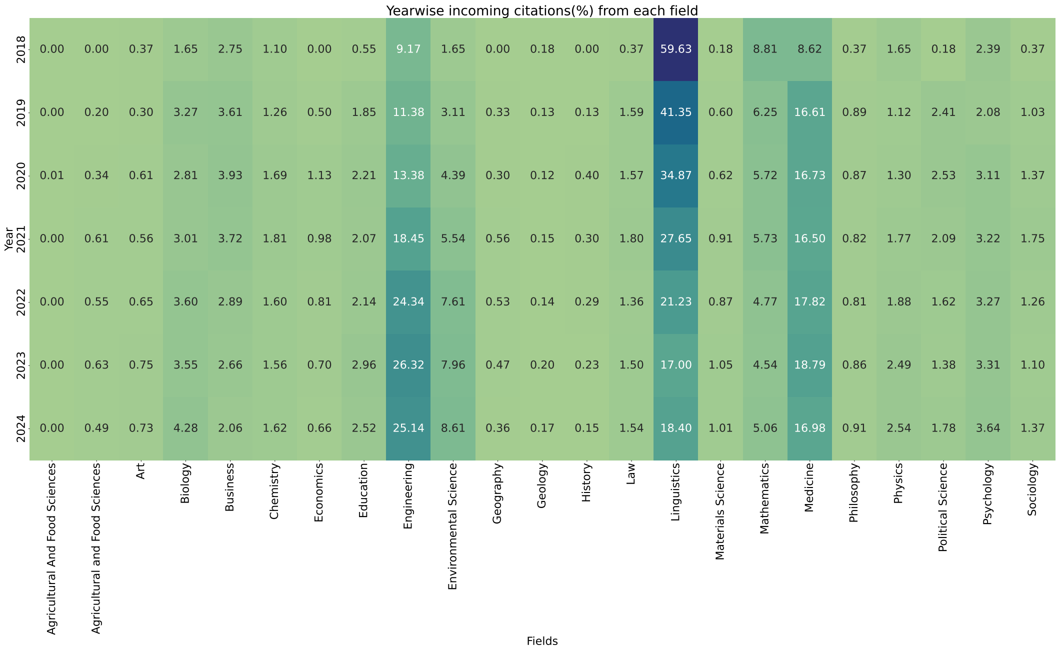

Fig 2. Yearwise Incoming LLM Citations (%).
