# Supplementary material for: Transforming scholarly landscapes: The influence of large language models on academic fields beyond computer science
Source: PLoS One. 2026 Jan 14;21(1):e0337127. doi: 10.1371/journal.pone.0337127 (PMC12893815; doi:10.1371/journal.pone.0337127)
Supplement: Attachment — Submitted filename: RebuttalLetter.docx [file pone.0337127.s004.docx]

Dear Reviewers,

We sincerely appreciate your constructive comments and feedback. Your feedback helped us clarify the scope of our contribution and strengthen the methodological transparency. We believe the revised manuscript is now more rigorous, balanced, and accessible. In this document, we explain the improvements that we brought in the present revision of the paper “*Transforming Scholarly Landscapes: The Influence of Large Language Models on Academic Fields beyond Computer Science.*”

Best Regards,

Authors

# **Reviewer #1**

## **Reviewer Concern 1:**

*Why were only traditional LLMs such as BERT included, while more recent architectures(e.g., instruction-tuned or multimodal models) are excluded, especially considering their growing adoption? Is the selection of LLMs skewed toward older or better-documented models? Are recent instruction-tuned or domain-specific LLMs underrepresented?*

**Response:**

We appreciate this important observation. Our dataset of 106 LLMs was curated from the Stanford CRFM Ecosystem Graphs, focusing on transformer-based foundation models with more than 100 million parameters that had identifiable academic publications and were pretrained on textual data as of February 2024. This criterion ensured that our analysis would be both reproducible and longitudinally consistent across the 2018-2024 timeframe.

As a result, the curated set naturally emphasized well-established and better-documented models such as BERT. Some of the more recent instruction-tuned, multimodal, or domain-specific LLMs were excluded because: (a) they lack a clear associated publication, (b) their bibliometric footprint is still limited or uneven across databases, and (c) their short citation history makes them unsuitable for trend analysis. We acknowledge this in both the “Research Methodology” and “Threats to Validity” sections.

## **Reviewer Concern 2:**

*Why were only 22 fields selected, and what criteria guided this selection? While these fields do reflect diverse applications, the limitation needs further justification.*

**Response:**
The 22 fields analyzed in our study are those provided by Semantic Scholar’s S2FOS3 field-of-study classifier, which assigns papers to one or more disciplinary categories with a reported accuracy of approximately 86%. These 22 fields span the natural sciences, social sciences, and humanities, covering the vast majority of non-CS citations to the LLMs.

## **Reviewer Concern 3:**

*Is citation count a sufficient and reliable proxy for measuring actual influence or usage of LLMs within a field? How are citation intents (e.g., methodological adoption vs. general reference) distinguished?*

**Response:**

This study investigates the citational influence of LLMs, which is important because citation remains the primary mechanism for acknowledging and crediting prior ideas in contemporary scientific research.

We recognize that research influence can extend beyond citations - for instance, when ideas are applied in practice without resulting in published work. However, such forms of impact fall outside the scope of this analysis.

The ways in which LLMs influence research extend beyond their direct use. For instance, LLM-related ideas may shape methodological choices or affect how studies position themselves within prior literature. In this paper, our primary focus is on the broad citational influence of LLMs. In addition, we gain partial insights into actual LLM usage through the analyses described below.

a. We analyzed **citation contexts** (sentences mentioning LLMs) and extracted task-related trigrams to better understand how LLMs are applied.
b. We conducted **manual validations** of these heuristics, confirming that the majority of extracted mentions reflected genuine uses.
c. For ethical-risk analysis, we validated keyword-based detection and found high precision and recall.

## **Reviewer Concern 4:**

*Are all citations given equal weight regardless of field norms or citation behaviors? For instance, fields like medicine or law have very different citation cultures compared to computer science or linguistics.*

**Response:**
We agree that citation practices differ substantially across disciplines: for example, biomedical sciences often produce papers with higher citation densities, while fields like law or philosophy cite more sparingly. To address this, we reported both absolute citation counts and field-normalized measures (i.e., the proportion of papers in each field citing LLMs). This normalization reduces disparities caused by field size and citation intensity, offering a fairer basis for comparison.

It is important to note that measuring the *exact degree of influence of individual citations* is extremely difficult and very possibly intractable. The perceived importance of the same two citations may differ across authors: one may view citation *A* as more influential than *B*, while another may see *B* as more central. Moreover, perceptions of influence vary by citation type. For example, some may consider a dataset citation highly influential, while others may place greater weight on methodological references. Similarly, path-breaking work may be seen either as foundational, without which the current study would not exist, or as a historical milestone now cited mainly out of convention rather than direct intellectual influence.

Given these complexities, our study does not attempt to assign varying weights to individual citations. Instead, we treat all citations equally and interpret them as *uniform indicators of engagement* with LLM-related research.

## **Reviewer Concern 5:**

*Does the paper account for global or linguistic disparities in LLM adoption across fields?*

**Response:**
We acknowledge this as an important limitation. Our dataset is drawn from Semantic Scholar (S2), which primarily indexes publications in English-language and Western venues. As a result, research outputs from non-English-speaking regions or locally published journals are likely underrepresented. This means our analysis reflects adoption patterns primarily in the globally indexed, English-dominant scientific literature, and may not capture the full extent of LLM usage in other linguistic or regional contexts. We have clarified this in the Threats to Validity section, noting that global and linguistic disparities are a limitation of our current study.

## **Reviewer Concern 6:**

*The paper uses co-authorship with CS-affiliated authors as a proxy for interdisciplinarity. This may be misleading, since many researchers in fields like engineering or linguistics have CS backgrounds, dual appointments, or publish in CS venues.*

**Response:**
We agree with the reviewer that affiliation-based proxies are a limited and imperfect measure of interdisciplinarity. However, our choice to use affiliation data was guided by two considerations:

1. Scalability and reproducibility: This data is consistently available in Semantic Scholar at a large scale, making it feasible to analyze ~148k citing papers. More nuanced measures, such as cross-field publication histories, are not yet reliably available at this scale.
2. Exploratory scope: The aim of our study is to provide a baseline descriptive mapping rather than a definitive measure of interdisciplinarity. In this context, co-authorship with CS-affiliated individuals serves as a first-order approximation of cross-field interaction.

We acknowledge in the revised manuscript (Research Methodology and Threats to Validity) that this metric should be interpreted with caution and not as a precise indicator of interdisciplinarity.

Note also that proxies are a way of gaining insight into phenomena that are difficult to directly ascertain. In such situations, it is beneficial to gain insights through multiple proxies. Our work provides one such proxy. We hope that future work will also explore other possible proxies. This will enable a greater understanding of the phenomenon. This is similar to how many studies with results pointing in the same direction inspire confidence in the conclusions. Each individual study is a valid research contribution that eventually enables that.

# **Reviewer #2**

## **Reviewer Concern 1:**

*The paper’s contribution seems mainly descriptive, relying on citation counts and Gini indices. Should it be reframed as a descriptive exploratory study rather than presented as a transformative impact analysis?*

**Response:**

We explicitly note in the introduction section and also highlight throughout the paper that our results should be interpreted as descriptive indicators of adoption and engagement, not as conclusive evidence of methodological uptake or impact.

## **Reviewer Concern 2:**

*The methodology relies only on descriptive statistics, without inferential testing. Task extraction and ethical-risk detection are not systematically validated. How do you ensure statistical reliability and reproducibility?*

**Response:**

The aim of this work is to establish a baseline map of LLM diffusion across fields, rather than test hypotheses about causality or statistical significance. We have clarified this scope in the revised abstract, introduction, and discussion sections.

At the same time, we strengthen the methodological transparency and reliability of our approach:

- For **task extraction**, we manually validated 100 representative trigrams and found that over 80% corresponded to genuine task descriptors.
- For **ethical-risk detection**, we manually evaluated 100 flagged papers and 200 random unflagged papers, confirming high recall (only seven papers missed) and perfect precision.
- For **fine-tuning vs. inference detection**, manual validation on 150 papers showed precision rates of 86% (fine-tuning) and 79% (inference).

These evaluations, now reported in the Research Methodology and Threats to Validity sections, provide reproducible precision/recall benchmarks for our heuristics.

## **Reviewer Concern 3:**

*What is the difference between “# papers citing LLMs” and “# citations to LLMs” in Table 1?*

**Response:**

- **# papers citing LLMs** refers to the number of unique non-CS research papers (∼148k) that cite at least one of the 106 curated LLM papers. Each paper is counted once, regardless of how many LLMs it cites.
- **# citations to LLMs** (∼273k) refers to the total number of individual citation instances from those papers to the curated LLM set. A single paper may contribute multiple citations if it references more than one LLM (e.g., citing both BERT and GPT-3), or if it cites the same LLM in different sections.

Thus, “# papers citing LLMs” measures breadth of engagement (how many papers reference LLMs at all), while “# citations to LLMs” measures intensity of engagement (how many times LLMs are cited in total across papers).

# **Other Updates**

- We added a new **Research Methodology** section, which consolidates data collection, model selection, field classification, analytical procedures, and heuristic validation. This makes the methodological choices explicit and transparent.
- We added a dedicated **Threats to Validity** section that discusses biases in citation data, disciplinary citation norms, model selection, field classification accuracy, global/linguistic disparities, interdisciplinarity measures, and heuristic reliability.
- We added a **Key Messages** subsection at the end of the Discussion, summarizing the main findings in a concise, reader-friendly format.
